# Histone Methyltransferase ASH1 Orchestrates Fibrogenic Gene Transcription During Myofibroblast Transdifferentiation

**DOI:** 10.1002/hep.25754

**Published:** 2012-08-28

**Authors:** Maria Jesus Perugorria, Caroline L Wilson, Mujdat Zeybel, Meagan Walsh, Shilu Amin, Stuart Robinson, Steven A White, Alastair D Burt, Fiona Oakley, Hidekazu Tsukamoto, Derek A Mann, Jelena Mann

**Affiliations:** 1 Institute of Cellular Medicine, Faculty of Medical Sciences, Newcastle UniversityNewcastle upon Tyne, UK; 2 Southern California Research Center for ALPD and Cirrhosis, Keck School of Medicine of the University of Southern California, Department of Veterans Affairs Greater Los Angeles Healthcare SystemLos Angeles, CA, USA

## Abstract

Transdifferentiation of hepatic stellate cells (HSCs) to a myofibroblast-like phenotype is the pivotal event in liver fibrosis. The dramatic change in phenotype associated with transdifferentiation is underpinned by a global change in gene expression. Orchestrated changes in gene expression take place at the level of chromatin packaging which is regulated by enzymatic activity of epigenetic regulators that in turn affect histone modifications. Using expression profiling of epigenetic regulators in quiescent and activated primary HSCs we found a number of histone methyltranferases including MLL1, MLL5, Set1 and ASH1 to be highly up-regulated during transdifferentiation of HSCs. All of these histone methyltranferases regulate methylation of lysine 4 of histone H3, which is a signature of actively transcribed genes. We therefore postulated that one or more of these enzymes may be involved in positively influencing expression of profibrogenic genes. *Conclusion:* We find that ASH1 directly binds to the regulatory regions of alpha smooth muscle actin (αSMA), collagen I, tissue inhibitor of metalloproteinase-1 (TIMP1) and transforming growth factor beta1 (TGFβ1) in activated HSCs while depletion of ASH1 caused broad suppression of fibrogenic gene expression. We also discovered that MeCP2 positively regulates ASH1 expression and therefore identify ASH1 as a key transcriptional activator component of the MeCP2 epigenetic relay pathway that orchestrates coordinated induction of multiple profibrogenic genes. (Hepatology 2012;56:1129–1139)

Myofibroblasts are the key cell type implicated in development of liver fibrosis.^1^-^3^ The vast majority of myofibroblasts in the injured liver are generated by way of transdifferentiation of resident hepatic stellate cells (HSCs).^1^ In normal liver, HSCs are quiescent, vitamin A-storing adipogenic cells; however, upon liver injury they undergo a major change in phenotype to become a myofibroblast.^2^ Such a dramatic phenotypic shift is underpinned by a global change in gene expression.^4^ Although many genes are down-regulated, there are a large number of up-regulated genes including proinflammatory and profibrogenic genes that synergistically drive fibrogenesis.^1^, ^2^, ^4^

Regulation of gene expression is an epigenetically governed process controlled by changes in chromatin structure.^5^, ^6^ Chromatin is a nucleocomplex comprising DNA and associated proteins, namely, histones. The chromatin structure can be modified through covalent modification to either the DNA or the histones, which in turn determines accessibility and recruitment of transcription factors and RNA polymerase II to the DNA.^5^, ^6^ We have recently described an MeCP2-dependent epigenetic pathway that facilitates myofibroblast transdifferentiation (MTD) of HSC by regulating the silencing of genes such as peroxisome proliferator-activated receptor gamma (PPAR-γ), which oppose MTD.^7^ MeCP2 has been shown to exert the same role during MTD in lungs, heart, and liver, suggesting that it is a conserved or “core” fibrogenic regulator operating in different organs to initiate fibrogenic responses to tissue injury.^7^-^9^ Epigenetic regulation can be exerted by way of three mechanisms; DNA methylation, noncoding RNA, and histone modifications.^10^-^14^ Histone modifications entail attachment of various functional groups (methyl, acetyl, ubiquitin, phospho, and sumo moieties among others) to defined residues within core histones.^10^, ^11^, ^13^ Combination of these modifications gives rise to the “histone code,” a language in which numerous signals emanating from covalent attachments to histone tails can instruct, coordinate, and finely tune gene expression. Given the complexity of epigenetic control of gene expression it is likely that a number of chromatin-modifying proteins will have critical functions in MTD, such as enzymes regulating specific histone modifications. Previous reports have studied the role of histone acetylation in HSC; however, these were not comprehensive studies and they did not explore the other forms of histone modification.

Methylation of lysine residues is probably the best studied of all histone modifications. It is now known that a large number of epigenetic enzymes carry out attachment of methyl groups to several lysines within histones, including lysines 4, 9, 27, 36, and 79 of histone H3 and lysine 20 of histone H4.^15^ Depending on the lysine that is modified, the presence of a methyl group can lead to gene activation (e.g., methylation of lysine 4 on histone H3), or gene repression (e.g., methylation of lysine 27 on histone H3).^16^, ^17^ Using *in vitro* and *in vivo* profiling of factors involved in epigenetic regulation, in this study we identify absent, small, or homeotic disc 1 (ASH1), a proven histone methyltransferase that targets lysine 4 on histone H3,^18^-^20^ as a positive regulator of multiple profibrogenic gene loci including transforming growth factor beta1 (TGF-β1), tissue inhibitor of metalloproteinase-1 (TIMP-1), and collagen I. Furthermore, we show that ASH1 is a key component of the previously described MeCP2 epigenetic relay pathway.^7^, ^8^

## Materials and Methods

### Human Subjects

The use of human tissue for scientific research was approved by Newcastle and North Tyneside Local Research Ethics (approval number 2003/26). All samples were collected subject to informed patient consent in writing.

### Isolation of Primary Human HSC

Primary human HSCs were isolated from normal margins of surgically resected liver. Liver tissue was digested with pronase and collagenase B (Roche) and the cell suspension was subsequently separated by an 11.5% Optiprep gradient (Sigma). HSCs were seeded onto plastic (Corning), cultured in Dulbecco's modified Eagle's medium (Life Technologies) supplemented with 16% fetal bovine serum, pyruvate, glutamine, penicillin, and streptomycin (Life Technologies) and maintained in an incubator at 37°C with 5% CO_2_. Cells at day 1 of *in vitro* culture were treated as quiescent and day 10 cultures were regarded as myofibroblasts (MFBs).

### Cell Isolation and Culture

Rat HSCs were isolated from normal livers of 350 g Sprague-Dawley rats by sequential perfusion with collagenase and pronase, followed by discontinuous density centrifugation in 11.5% Optiprep (Invitrogen). HSC from chronic carbon tetrachloride (CCl_4_)-injured livers, bile duct ligation (BDL) livers, and controls were isolated as described.^7^ Briefly, activated HSCs were isolated from rat livers injured by BDL for 10 days or twice weekly repeated injury with CCl_4_ for 3 weeks. Mouse HSCs were isolated from C57Bl6 using sequential pronase/collagenase digestion followed by Nycodenz density-gradient centrifugation as described.^21^ Purity of HSC preparations was assessed by autofluorescence 1 day after isolation and was always higher than 97%. Mouse HSCs were isolated from *Mecp2*^−/*y*^ livers as described.^7^ Rat and mouse HSCs were cultured on plastic in Dulbecco's modified Eagle's medium, supplemented with 100 units/mL penicillin, 100 μg/mL streptomycin, 2 mM L-glutamine, and 16% fetal calf serum. Cell cultures were maintained at 37°C at an atmosphere of 5% CO_2_. Freshly isolated (day 0) cells were considered quiescent and day 10 cultures regarded as MFBs unless specifically stated otherwise.

### Animals

*Mecp2*^−/*y*^ mice were obtained from Jax labs (strain B6.129P2(C)-Mecp2tm1.1Bird/J) and are a cross of a constitutive CMVCre strain and a strain carrying *mecp2* gene containing loxP sites around exons 3 and 4. Authors hold appropriate licenses for animal experiments, which were issued/approved by the local ethical committee and the UK Home Office.

### Chronic CCl_4_ Liver Injury Model

Fibrogenesis was induced by 3-week CCl_4_ treatment of 6-week-old *mecp2*^−/*y*^ knockouts or age-matched wildtype (WT) littermates. Mice were injected intraperitoneally twice weekly with CCl_4_/olive oil in a 1:1[vol/vol] ratio at 1 μL/g body weight. Twenty-four hours after the final CCl_4_ administration, animals were sacrificed and liver samples prepared.

### Immunohistochemistry

Formalin-fixed sections were dewaxed in clearene and dehydrated in alcohol. Antigen retrieval and nonspecific blocking was as described.^7^ Slides were incubated overnight at 4°C with anti-ASH1 (ab4477, Abcam) at a 1:100 dilution in phosphate-buffered saline (PBS) containing 20% swine serum, then washed in PBS and incubated with biotinylated swine antirabbit at 1:200 (Dako) for 2 hours. After PBS washing slides were incubated with streptavidin biotin-peroxidase complex (Vector Laboratories) and incubated at room temperature for 45 minutes. ASH1-positive cells by were visualized by 3,3′-diaminobenzidine tetrahydrochloride (DAB).

### Small Interfering RNA (siRNA) Transfection

Rat MFBs generated by culturing HSCs for 10 days on plastic were transfected with siRNA designed to silence either rat ASH1 (catalog numbers s160506 and s160508) or negative control siRNA (catalog number 4390844, all from Ambion). siRNAs were transfected into 5 × 10^6^ rat MFB using a square wave electroporator BTX830 (Harvard Apparatus) set at 500V to deliver three pulses of 10 ms (full description^7^). Briefly, MFBs were trypsinized, washed once, and resuspended in 700 μL serum-free media, incubated with a total of 2 μg siRNA, and current applied. The cells were allowed to grow for 48 hours posttransfection, when they were harvested and RNA and/or whole cell extracts made.

### Sodium Dodecyl Sulfate-Polyacrylamide Gel Electrophoresis (SDS-PAGE) and Immunoblotting

Total protein was fractionated by 7–12% SDS-PAGE and transferred to nitrocellulose membrane. Blots were blocked with TBS/Tween 20 (0.1% T-TBS) containing 5% milk protein before overnight incubation with primary antibodies. Primary antibodies raised against ASH1 (ab4477 and ab50981, Abcam), MLL5 (ab75339, Abcam), Total Histone H3 (9715, Cell Signaling), Histone H3 dimethyl K4 (H3K4me2, ab32356, Abcam), Histone H3 trimethyl K36 (H3K36me3, ab9050-100, Abcam), Set 1 (ab70378, Abcam), Histone H3 trimethyl K4 (H3K4me3, ab8580, Abcam), Histone H3 trimethyl K9 (H3K9me3, ab8898, Abcam), Histone H3 dimethyl K27 (H3K27me2, ab24684, Abcam), Histone H3 trimethyl K27 (H3K27me3, CS-069-100, Diagenode); α-smooth muscle actin (αSMA, A5228, Sigma), TIMP-1 (sc-6834, Santa Cruz Biotechnology), and Collagen Type I (600-401-103, Rockland Immunochemicals) were used at 1:1,000 dilution and β-actin (A5316, Sigma) was used at 1:2,000 dilution. Membranes were washed in T-TBS and incubated with antirabbit (7074S, Cell Signaling), antimouse (A4416, Sigma), or antigoat (A5420, Sigma) horseradish peroxidase (HRP)-conjugate antibodies at 1:5,000 dilution for 1 hour. Blots were washed and antigen detected by ECL (Amersham Biosciences).

### Quantitative Reverse Transcriptase-Polymerase Chain Reaction (qRT-PCR)

Total RNA was purified from isolated cells using the Total RNA purification kit (Qiagen, UK) following the manufacturer's instructions. One microgram isolated total RNA was DNAse treated (Promega) and used as template to generate complementary DNA (cDNA) utilizing a random hexamer primer [p(dN)6] and MMLV reverse transcriptase (Promega). Primers for Procollagen I, α-SMA, TIMP-1, and TGF-β-1 were as reported.^21^ Primers for histone lysine methyltransferases and histone lysine demethylases are included in Supporting Table 1. Quantitative PCR program: 20 seconds at 94°C, then 40 cycles of 5 seconds at 94°C, 20 seconds at 55°C, 30 seconds at 72°C, followed by ABI7500 machine predetermined melt curve. All reactions were normalized to the internal control and relative level of transcriptional difference calculated using the following equation: (1/(2A)) ×100.

### Crosslinked Chromatin Immunoprecipitation (XChIP) Assay

A ChIP assay was carried out using 100 μg crosslinked chromatin prepared from rat HSC or MFB as described.^7^ Antibodies used for immunoprecipitation raised against ASH1 (ab4477), MLL5 (ab75339), and Histone H3 tri methyl K4 (ab8580) were all purchased from Abcam. Ten μg of each antibody or appropriate irrelevant antibody control were used in each ChIP reaction. Primers used for detection of relevant rat genomic promoter sequences were: TGF-β1 sense 5′-aagaaacgcctctctgtcca-3′ and antisense 5′-caggtcagctgggctacatt-3′; TIMP-1 sense 5′-ctctgccacccctcacca-3′ and antisense 5′-ggactggatgggcctcgt-3′; collagen I sense 5′-ggggacaagggtggcagaa-3′ and antisense 5′-gaggagggctgggaggaacc-3′. Each PCR reaction was performed in triplicate and the analysis was repeated three times from independent ChIP experiments. A signal intensity value for each sample was calculated from the average of the experiments. Average values of eluates were normalized to average values of control antibody sample and expressed as fold enrichment above background (i.e., control antibody).

## Results

**Fig. 1:**
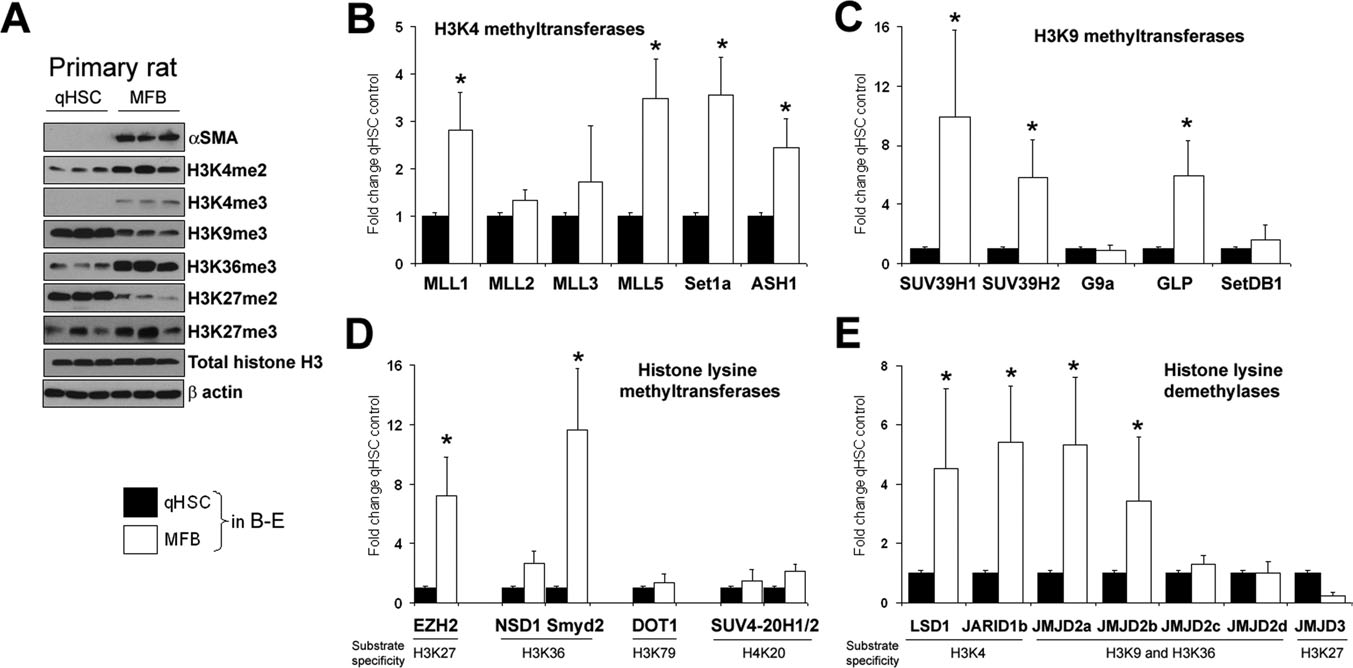
Expression profile of epigenetic regulators in quiescent and activated HSCs. (A) Ten μg whole cell protein extract from three separate preparations of primary rat qHSCs (culture day 1) or activated HSCs (culture day 10) were separated by SDS-PAGE and immunoblotted for αSMA, H3K4me2, H3K4me3, H3K9me3, H3K36me3, H3K27me2, and H3K27me3 histone modifications. Equal loading was assessed using total histone H3 and β-actin controls. (B-E) Messenger RNA (mRNA) levels of histone lysine methyltransferases and demethylases were quantified by qRT-PCR in three separate preparations of primary rat qHSCs (day 0) and day 10 transdifferentiated myofibroblasts. Alternative names for listed enzymes are as follows: SUV39H1 (KMT1A), SUV39H2 (KMT1B), G9a (KMT1C), GLP (KMT1D), SETDB1 (KMT1E), MLL1 (KMT2A), MLL2 (KMT2B), MLL3 (KMT2C), MLL5 (KMT2E), SET1a (KMT2F), NSD1 (KMT3B), ASH1 (KMT2H), SMYD2 (KMT3C), DOT1 (KMT4), EZH2 (KMT6), SUV4-20H1 (KMT5B), SUV4-20H2 (KMT5C), LSD1 (KDM1), JMJD2A (KDM4A), JMJD2B (KDM4B), JMJD2C (KDM4C), JMJD2D (KDM4D), JARID1B (KDM5B), JMJD3 (KDM6B). Error bars represent mean values ± standard error of the mean (SEM). *P < 0.05.

**Fig. 2:**
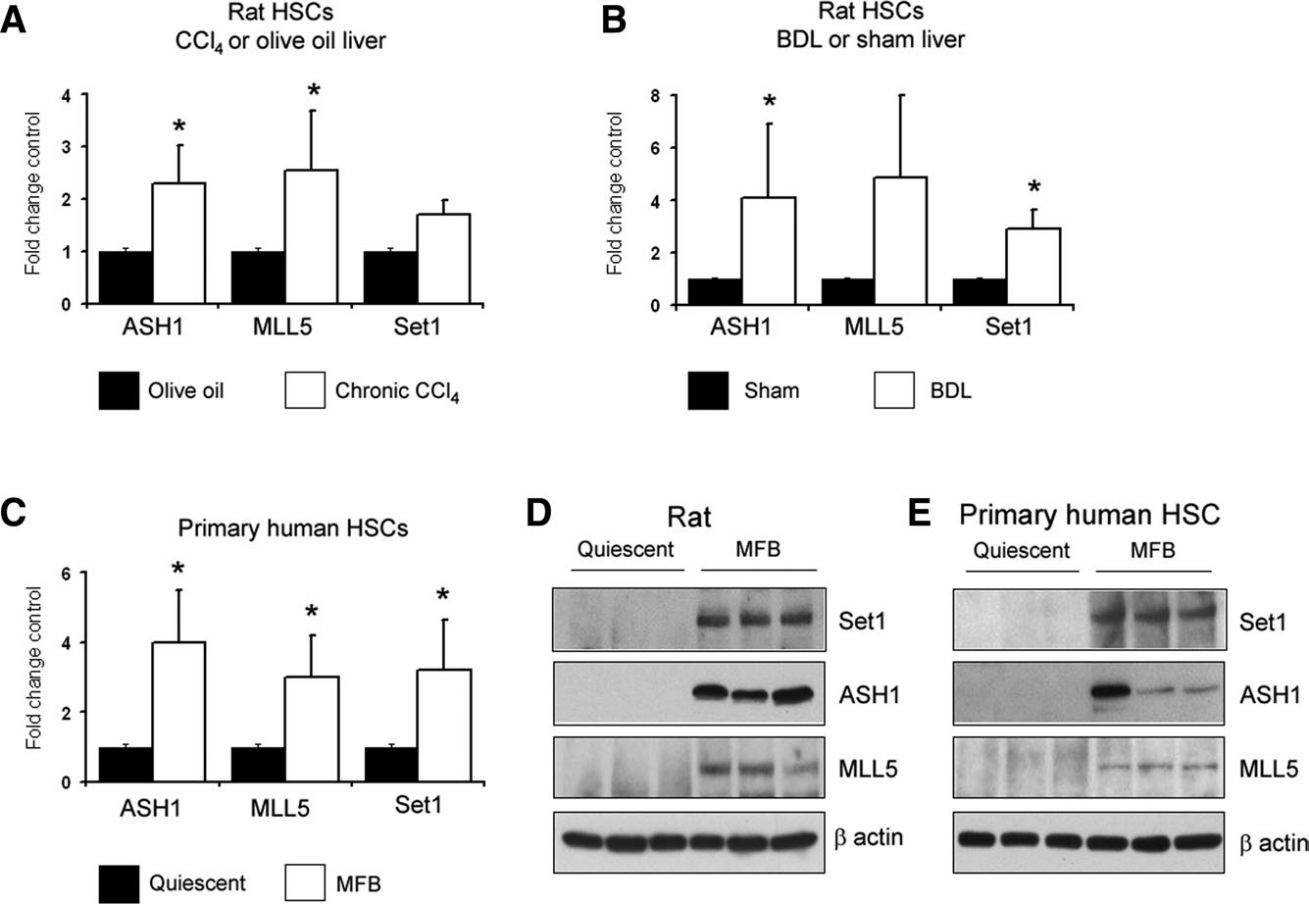
Expression of ASH1, Set1, and MLL5 histone methyltransferases is highly up-regulated during the MTD of HSCs. (A-C) qRT-PCR analysis of ASH1, Set1, and MLL5 mRNA was performed using RNA extracted from HSCs isolated from CCl_4_ (or olive oil) and BDL (or sham operated) injured rat livers (n = 5) or primary human qHSCs or activated HSCs. Error bars represent mean values ± SEM. *P < 0.05. (D) Thirty μg whole cell protein extract from three separate preparations of rat and (E) primary human qHSC or activated myofibroblasts were separated by SDS-PAGE and immunoblotted for Set1, ASH1, MLL5, and β-actin.

MTD of HSCs is caused by a global expression change in hundreds of genes, a process that is at least in part regulated epigenetically.^7^ Numerous enzymes regulate histone methylation and we postulated there may be changes in expression of these epigenetic regulators as well as their target modifications during MTD. We initially assessed global levels of histone methylation signatures in quiescent versus *in vitro* transdifferentiated rat HSCs and discovered major shifts in almost all of the histone marks tested (Fig. 1A). Histone marks associated with active transcription, namely, dimethylated and trimethylated H3K4 as well as trimethylated H3K36, are all increased in myofibroblasts as compared to quiescent HSC. Alteration in global levels of repressive histone marks were found to be mixed, with trimethylated H3K9 and dimethylated H3K27 being down-regulated, whereas trimethylated H3K27 was increased. We also show αSMA levels in all samples as confirmation of HSC MTD (Fig. 1A). We next tested for expression levels of epigenetic enzymes that regulate histone modifications; these were grouped based on the lysine residue that is the modifying target (Fig. 1B-E). Most of the enzymes were shown to increase in their expression, with only a small number remaining unchanged or down-regulated. We were particularly intrigued to discover almost all of the H3K4 methyltransferases (HMTases) to be up-regulated with MTD (Fig. 1B); these enzymes regulate attachment of methyl groups to lysine 4 on histone H3, an epigenetic mark known to positively regulate transcriptional activation of genes. Interestingly, most of the histone lysine demethylases also increase with MTD, particularly those regulating demethylation of H3K4, suggesting that regulation of this histone mark is likely regulated by a balance of two enzyme sets with opposing actions. To confirm trends in expression levels of H3K4 HMTases and a select number of other epigenetic enzymes shown in Fig. 1A, RNA was isolated from primary human HSCs or myofibroblasts as well as *in vivo* activated HSCs from BDL/sham or chronic CCl_4_/olive oil control animal livers. We found that three of the H3K4 methyltransferases, namely, MLL5, Set1, and ASH1, increased in all models and species tested (Fig. 2A-C). Up-regulation of MLL5, Set1, and ASH1 protein expression during MTD was further confirmed in three separate preparations of both rat and primary human HSCs (Fig. 2D,E).

**Fig. 3:**
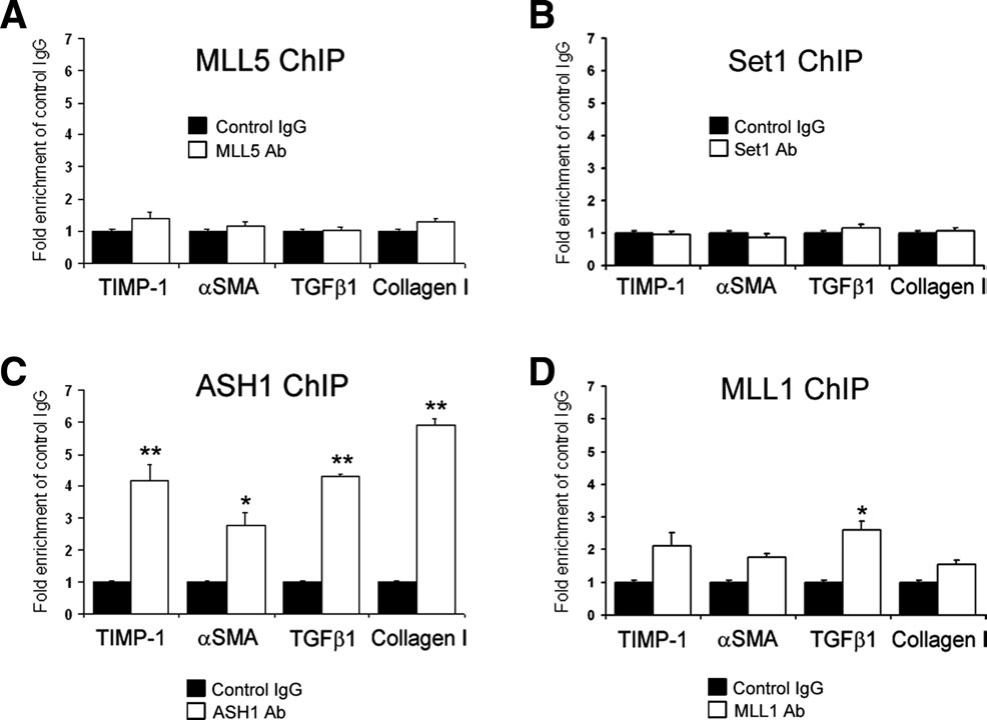
ASH1, but not MLL5 or Set1, binds to the regulatory regions of profibrogenic genes. One hundred μg of crosslinked chromatin from primary rat myofibroblasts was incubated with 10 μg of (A) anti-MLL5, (B) anti-Set1, (C) anti-ASH1, or (D) anti-MLL1 antibody and ChIP assay carried out. Immunoprecipitated DNA was used as template in qPCR reactions using primers specific for TIMP-1, αSMA, TGF-β-1, and Collagen I distal promoter regions (n = 3). *P < 0.05 and **P < 0.01.

**Fig. 4:**
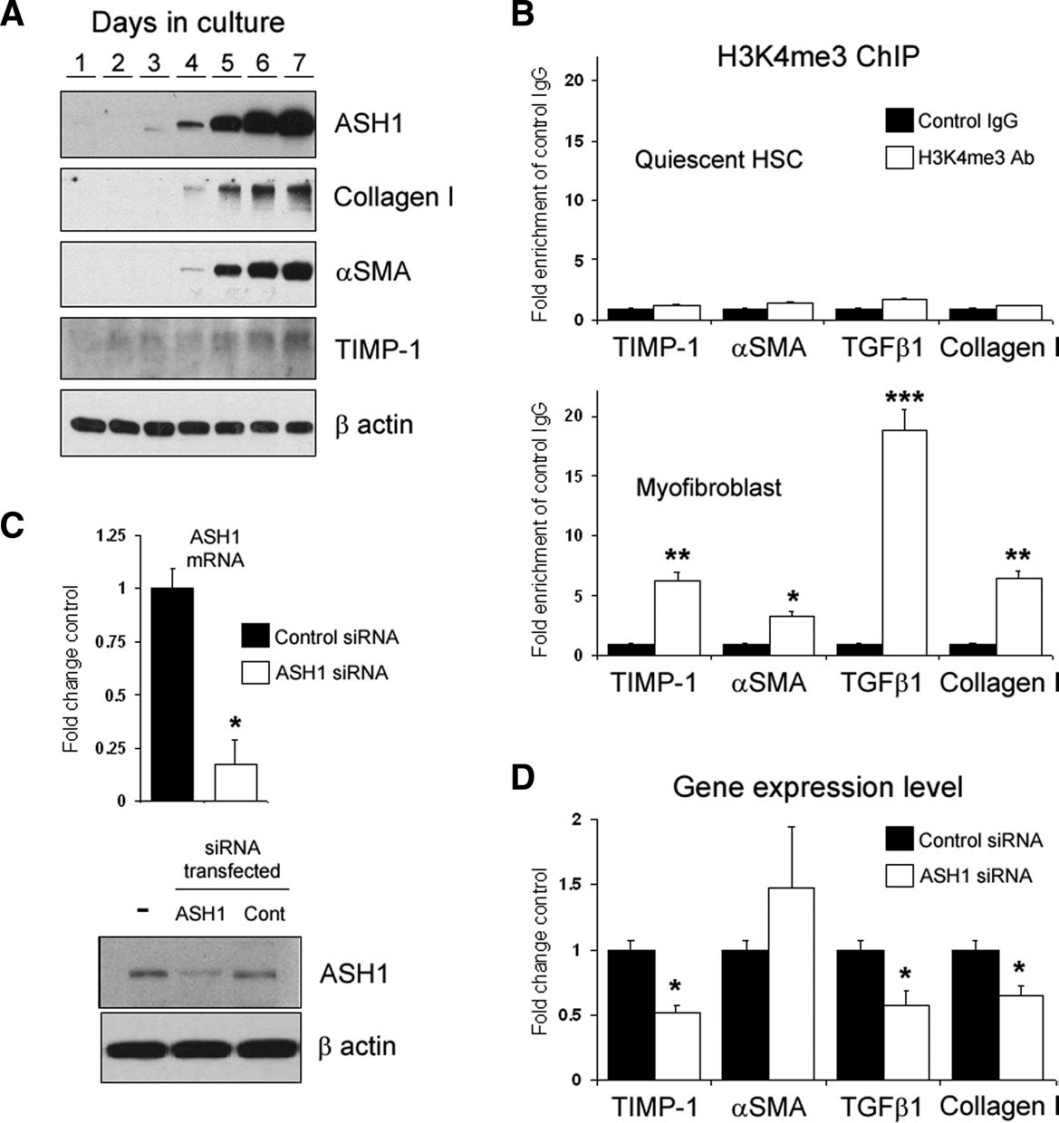
ASH1 positively influences the expression of TIMP-1, TGF-β-1, and Collagen I. (A) Fifty μg whole cell extract from rat HSCs harvested at days 1, 2, 3, 4, 5, 6, and 7 of *in vitro* culture were separated by SDS-PAGE and immunoblotted for ASH1, Collagen I, αSMA, TIMP-1, and β-actin. (B) One hundred μg of crosslinked chromatin from primary rat quiescent HSCs (top panel) or myofibroblasts (bottom panel) was incubated with 10 μg of anti-trimethyl H3K4 and ChIP assay carried out. Immunoprecipitated DNA was used as template in qPCR reactions using primers specific for TIMP-1, αSMA, TGF-β-1, and Collagen I distal promoter regions (n = 3). (C) 5 × 10^6^ rat myofibroblasts were electroporated with 2 μg of control or ASH1 siRNA. Cells were harvested 48 hours later, and RNA and protein isolated. qRT-PCR analysis of ASH1 expression was performed (top panel). Twenty μg whole cell protein extract were separated by SDS-PAGE and immunoblotted for ASH1 and β-actin (bottom panel) (representative gel shown, n = 3). (D) RNA isolated from myofibroblasts transfected with control or ASH1 siRNA was used for qRT-PCR analysis of TIMP-1, αSMA, TGF-β-1, and Collagen I (n = 3). Error bars represent mean values ± SEM. *P < 0.05, **P < 0.01, and ***P < 0.001.

Trimethylation of H3K4 at promoter and the 5′ end of the gene confers transcriptional activation of gene expression.^22^, ^23^ Profibrogenic genes, such as αSMA, TIMP-1, collagen I, and TGF-β1, are highly up-regulated with HSC MTD.^2^, ^4^ We therefore wondered if Set1, ASH1, or MLL5 may regulate transcriptional activation of profibrogenic genes during MTD. Using ChIP, we tested for direct binding of Set1, ASH1, and MLL5 to αSMA, TIMP-1, collagen I, and TGF-β1 genes. ASH1, but not MLL5 or Set1, bound to proximal promoter/5′ end of all four genes in primary, *in vitro* activated rat myofibroblasts (Fig. 3A-C). Previous studies have shown that ASH1 and MLL1, another HMTase that also regulates H3K4 trimethylation, are closely related and can have redundant functions in cells. We therefore also carried out a ChIP assay for MLL1 on the same genes (Fig. 3D). Only the TGF-β1 gene bound MLL1 in activated HSCs, albeit at a low level, suggesting that there may be some limited functional redundancy between ASH1 and MLL1 in these cells. If ASH1 positively regulates expression of profibrogenic genes, then its expression should correlate with expression of its target genes during MTD. Using protein extracts collected daily from initial isolation and during culture-driven activation of primary rat HSCs, we show that ASH1 expression is induced on day 4 of HSC activation, reaching a maximal level around day 15, when MTD is complete. The target genes collagen I, αSMA, and TIMP-1 were found to follow an almost identical trend (Fig. 4A).

ASH1 exerts its positive effect on gene transcription by increasing the level of lysine 4 trimethylation at histone H3 in gene promoter regions. We therefore assessed the amount of H3K4me3 in proximal promoter region of profibrogenic target genes where we detected ASH1 binding. Compared to quiescent HSCs, there was increased trimethylated lysine 4 at histone H3 associated with proximal promoter of collagen I, αSMA, TIMP1, and TGF-β1 genes in myofibroblasts, which was not detected in quiescent HSCs (Fig. 4B). To show that ASH1 regulates these genes, a cocktail of two siRNAs targeting ASH1 were transfected into myofibroblasts (Fig. 4C) and levels of TIMP1, αSMA, collagen I, and TGF-β1 transcripts examined (Fig. 4D). Reduction in ASH1 led to a significant decrease in target gene transcription, with the exception of αSMA, which appeared unaffected (Fig. 4D).

**Fig. 5:**
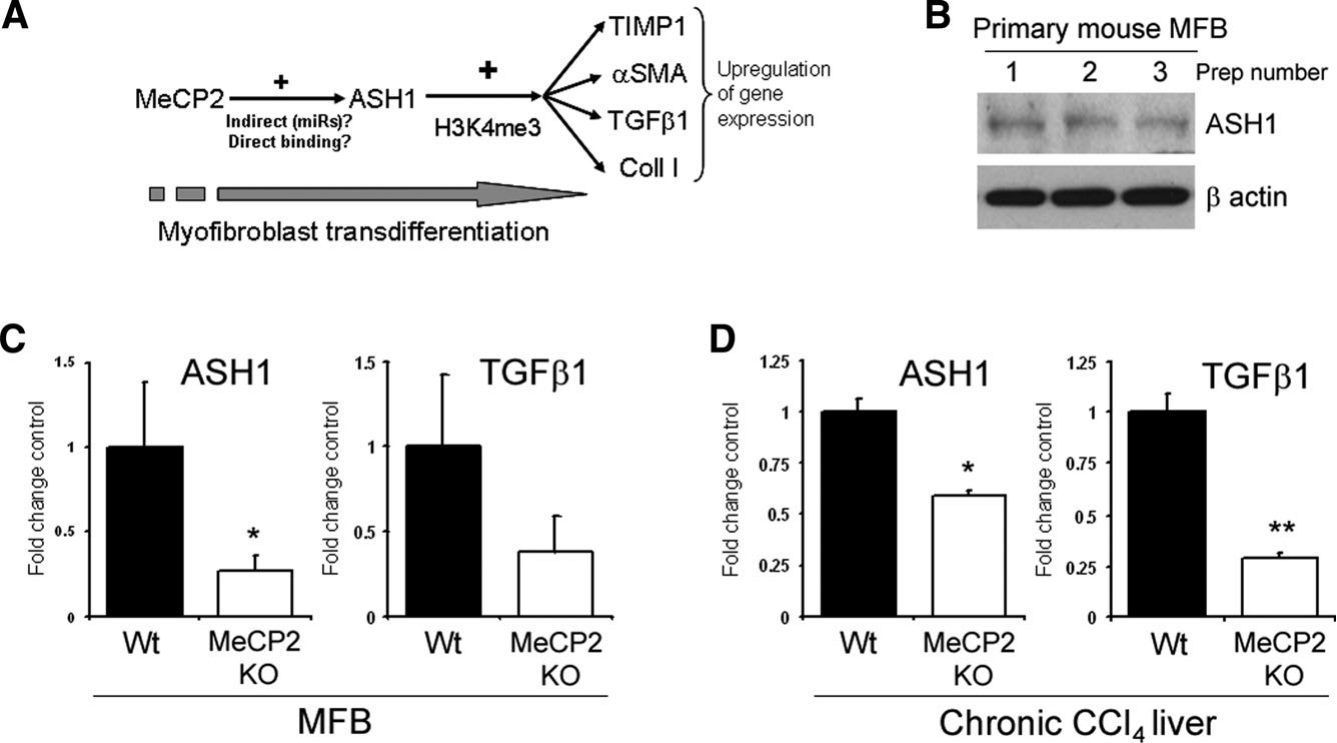
(A) MeCP2 and ASH1 operate in a sequential regulatory pathway that culminates in transcriptional activation of profibrogenic genes. (B) Twenty μg whole cell protein extract from three separate preparations of primary C57Bl6 mouse myofibroblasts were separated by SDS-PAGE and immunoblotted for ASH1 and β-actin. (C) qHSCs were isolated from WT or *Mecp2*^−/*y*^ KO livers and transdifferentiated *in vitro* for 14 days. Total RNA was prepared from both cell populations and qRT-PCR was carried out to analyze ASH1 and TGF-β-1 expression (n = 4). (D) RNA was isolated from chronic CCl_4_-injured WT or *Mecp2*^−/y^ liver and used in qRT-PCR reactions with primers specific for mouse ASH1 and TGF-β1 (n = 4). Error bars represent mean values ± SEM. *P < 0.05, **P < 0.01.

We were next interested to determine how ASH1 expression is regulated during HSC MTD. Based on the results in Figs. 1B and 2, ASH1 is at least in part regulated at the level of gene transcriptional, but possibly also by posttranscriptional mechanisms. Given the apparent pivotal role of MeCP2 in fibrogenesis we were interested to determine if it might contribute to the regulation of ASH1 expression (Fig. 5A). To test this idea, we prepared HSCs from WT and *mecp2*-deficient (*mecp2*^−/*y*^) mice and cultured them for 10 days to generate MFBs. ASH1 protein was expressed in primary WT myofibroblasts (Fig. 5B); however, its expression was significantly reduced in *mecp2*^−/*y*^ myofibroblasts (Fig. 5C, left panel). Furthermore, expression of the putative ASH1 target TGF-β1 was also significantly reduced in cells lacking MeCP2 (Fig. 5C, right panel). MeCP2 absence results in around 60% reduction in both collagen I and TIMP-1, as reported.^7^ ASH1 and TGF-β expression were also at reduced levels in liver tissue from chronic CCl_4_ injured *mecp2*^−/*y*^ mice (Fig. 5D).

**Fig. 6:**
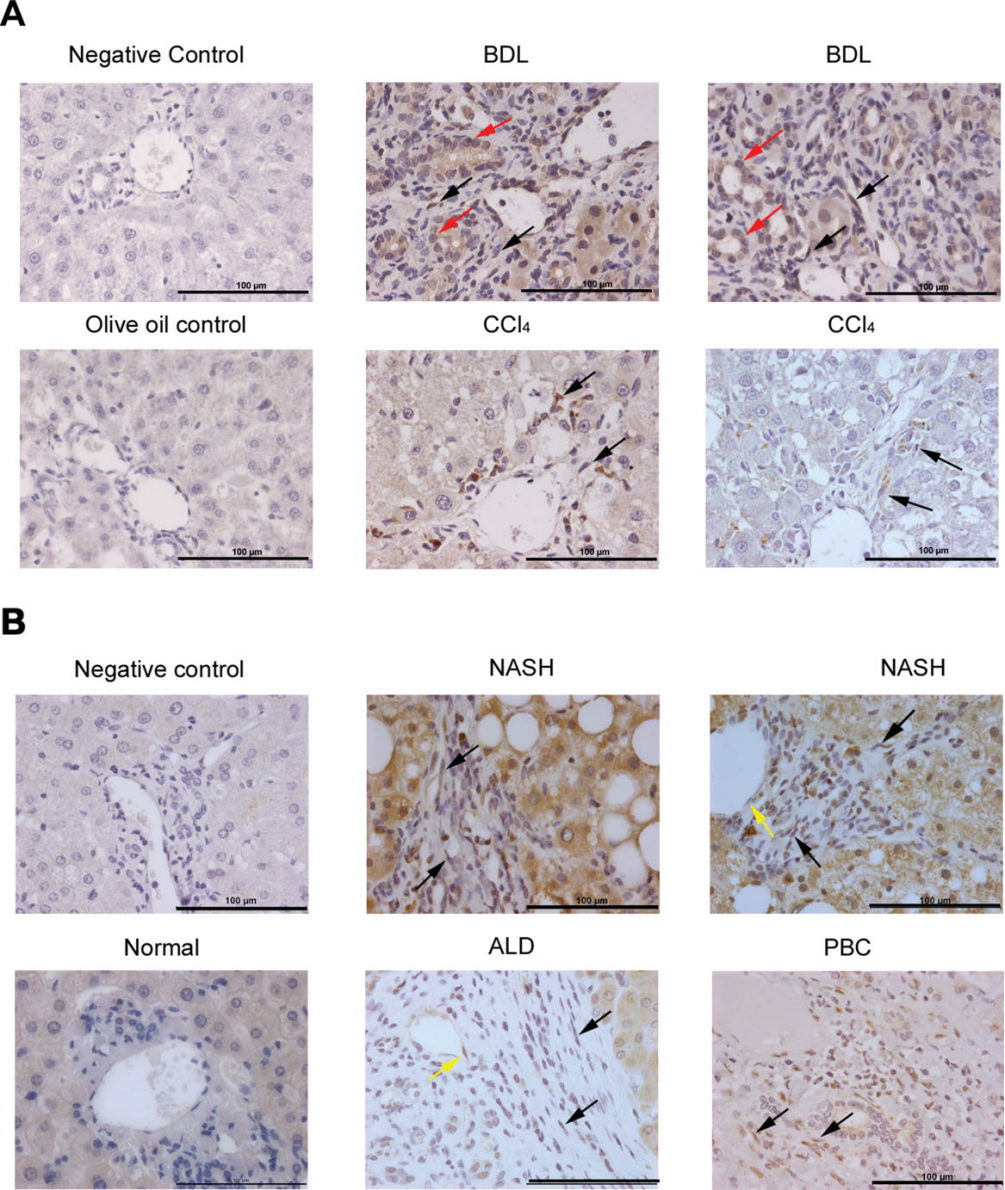
ASH1 is highly expressed in both rat and human liver disease. (A) Representative liver sections showing ASH1 immunohistochemical staining in either BDL rat liver (BDL), 4-week CCl_4_-injured rat liver, and normal control liver. (B) Photomicrographs depict liver sections from patients with NASH, ALD, and PBC or normal liver immunohistochemically stained with ASH1-specific antibodies. Black arrows denote ASH1-positive hepatic myofibroblasts, yellow arrows are indicative of positive endothelial cells, and red arrows show ASH1 expression in proliferating ductular cells. All photomicrographs are at 400× magnification.

In addition to *in vitro* evidence demonstrating the role of ASH1 in myofibroblasts, we carried out immunohistochemical staining of CCl_4_- and BDL-injured livers to confirm the significance and localization of ASH1 protein in animal models of liver fibrosis (Fig. 6A). CCl_4_-injured livers show strong ASH1 staining associated with myofibroblasts located within fibrotic tissue, whereas BDL livers show additional ASH1-positive staining in proliferating bile ducts (Fig. 6A). Furthermore, myofibroblasts in human livers of varied pathologies including alcoholic liver disease (ALD), nonalcoholic steatohepatitis (NASH), and primary biliary cirrhosis (PBC) also express ASH1. Occasionally, other nonparenchymal cells including endothelial cells or macrophages were positive in human disease. However, weaker ASH1 immunoreactivity was also observed within hepatocytes, both in normal and diseased livers (Fig. 6B).

## Discussion

Wound healing and consequent scar formation are crucial protective responses of organs to cellular damage. Myofibroblasts are the key cell type in wound healing, responsible for orchestration of matrix remodeling to form a scar, recruitment of inflammatory infiltrate, and wound contraction. During acute liver injury, myofibroblasts transiently appear until the wound is resolved. However, in chronic liver injury myofibroblasts persist, proliferate, and cause fibrosis. In order to avoid such pathogenic outcomes, the production of myofibroblasts has to be regulated by tightly controlled mechanisms. Although a plethora of knowledge exists relating to myofibroblast apoptosis and survival, we still have very little understanding of how myofibroblast MTD is mechanistically regulated. This study describes a novel H3K4 methyltransferase that augments activation of several profibrogenic genes known to be necessary for the onset and progression of fibrogenesis. Furthermore, these findings form a new addition to the previously described MeCP2 epigenetic relay that regulates myofibroblast MTD.^7^

Comparison of histone modifications between quiescent HSCs and myofibroblasts has shown that, even at a global level, significant shifts in the overall amount of various histone modifications are observed during MTD. These changes are orchestrated through altered expression and activity of numerous histone-modifying enzymes. In this study we focused on lysine methyltransferases (HMTases) and using an initial screen we identify Set1, ASH1, and MLL5 as three HMTases that are up-regulated in several models of MTD *in vitro* and *in vivo*, both in rat and primary human HSC. Although all three enzymes are able to regulate gene activation, it is only ASH1 that directly binds to TIMP-1, αSMA, collagen I, and TGF-β1 gene promoters and increases levels of associated histone H3 lysine 4 trimethylation. In addition, MLL1, an HMTase closely related to ASH1, appears to display some functional redundancy with ASH1, as shown by weak binding to the TGF-β1 gene promoter.

Expression of ASH1 increases during MTD and reinforces the transcription of target profibrogenic genes; its functional activity at these genes was confirmed using siRNA targeted ASH1 knockdown, which reduced expression of all profibrogenic genes, with the exception of αSMA. One possible explanation is that αSMA is controlled by more than one HMTase or that other compensatory/regulatory mechanisms may operate to maintain αSMA transcription constant in conditions where levels of ASH1 are reduced. Of note, the H3K4 demethylases that oppose action of ASH1 and other lysine HMTases are also up-regulated during MTD (Fig. 1E). It would be interesting to find out how, and at which genes, these two sets of enzymes exert their opposing roles. However, this is beyond the scope of the current study.

ASH1 is a member of trithorax group of proteins; it contains several domains including a SET domain responsible for mono-, di-, and tri-methylating lysine residues.^18^-^20^ The manner in which ASH1 activates transcription is thought to involve binding of chromatin remodeling complexes to deposited trimethylated H3K4; these include ATPases which inhibit interaction of epigenetic repressors, namely HP1, with chromatin. As such, the activity of ASH1 and recruited remodeling complexes contribute to the establishment of epigenetically active chromatin structures.^17^-^19^

**Fig. 7:**
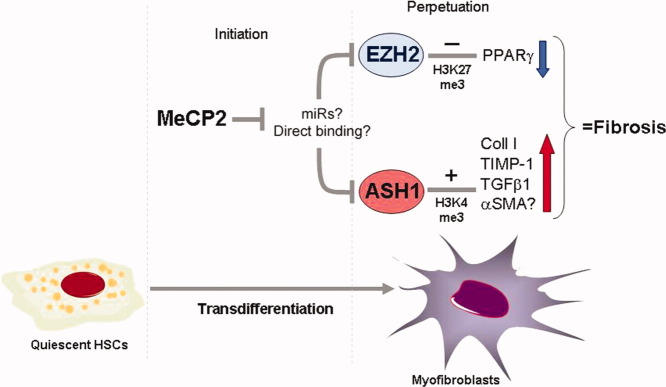
MTD-associated epigenetic regulatory pathways: MeCP2 positively regulates expression of EZH2 and ASH1 histone methyltransferases. These HMTases regulate methylation of H3K27 and H3K4, respectively. As such, EZH2 is inhibitory, whereas ASH1 activates gene expression. MeCP2 protein becomes expressed with the onset of MTD; it may then bind to one or more miRNA promoters causing their transcriptional repression. Reduction in these miRNAs may release a translational block on both EZH2 and ASH1, which in turn leads to repression of PPAR-γ (by EZH2 and MeCP2) and simultaneous activation of collagen I, TIMP-1, and TGF-β1 (by ASH1). Alternatively, MeCP2 could directly activate EZH2 and ASH1 expression.

How is ASH1 expression regulated in HSCs? Quiescent HSCs contain some ASH1 transcript which increases with MTD, but no detectable protein, suggesting that in addition to transcriptional control, ASH1 expression is at least in part regulated by protein translation. The ASH1 transcript is a putative target for miR137, which is a reported target for MeCP2 transcriptional repressor.^24^ MeCP2 is rapidly up-regulated in day 1 early activating HSCs, which in turn may block expression of miR137, thus releasing a translational block on ASH1. As expected from our hypothesis (Fig. 5A), the absence of MeCP2 in *mecp2*^−/*y*^ knockout myofibroblasts led to reduction of ASH1 expression. Chronically injured MeCP2 knockout livers are protected from fibrosis, which may be in part related to significantly reduced ASH1 expression and consequent diminution of profibrogenic genes collagen I, TIMP-1, and TGF-β1 (Fig. 5C,D).^7^ We have previously shown that MeCP2 positively regulates expression of EZH2 HMTase that controls methylation of H3K27 leading to gene repression.^7^ Interestingly, EZH2 is also a target for miR137.^25^ Induced EZH2 expression in myofibroblast and its targeting to 3′ end of PPAR-γ cause repression of this gene, thus aiding MTD.^7^ Therefore, this suggested pathway may at least in part explain how MeCP2 regulates HMTases that are both activators and repressors of transcription (for a schematic of the epigenetic pathway, see Fig. 7).

The data from our previous and current studies suggest that MeCP2 regulates both repression of antifibrogenic genes (by way of EZH2) and activation of profibrogenic genes (by way of ASH1), confirming its role as a pivotal regulator of HSC MTD (Fig. 7). However, it is likely that several microRNAs and/or other epigenetic modulators may form further, as yet undefined components of this mechanism. Equally, the existence of other, distinct and unrelated epigenetic mechanisms that regulate fibrosis should not be discounted.

The discovery of a new epigenetic regulator ASH1 in myofibroblast MTD along with its role as a vital part of previously described epigenetic relay with MeCP2 as a central component significantly furthers our understanding of the control of wound healing (Fig. 7). The role of ASH1 in diseased liver, however, is less clear. Although its expression is confirmed in myofibroblasts of diseased livers, basal expression is also evident in hepatocytes, particularly in human liver. Although previous reports rule out expression of MeCP2 in hepatocytes, we have recently detected low-level expression in mouse hepatocytes. This raises a possibility that ASH1 may be subject to control by MeCP2 in hepatocytes, although we cannot exclude the existence of cell-specific regulatory mechanisms. As ASH1 conditional knockout mice are not currently available to enable targeted deletion to either hepatocytes or myofibroblasts, we are not yet able to definitively address the *in vivo* role of ASH1 in fibrogenesis. Absence of selective pharmacological inhibitors of ASH1 also limits *in vivo* studies.

In summary, we describe ASH1 as a histone-modifying enzyme that functions as a regulatory component of the MeCP2-regulated fibrogenic pathway that remodels the epigenetic landscape of HSC during MTD. Further defining how MeCP2 and ASH1 cooperate to remodel histone structure at specific fibrogenic genes will be important for determining if and how ASH1 activities may be targeted for therapeutic applications. It will also be of great interest to examine the role of ASH1 in other organ systems to discover if, like MeCP2, it is a conserved component of the fibrogenic process.

## Abbreviations

>ASH1: absent, small, or homeotic disc 1
BDL: bile duct ligation
ChIP: chromatin immunoprecipitation
H3K4: lysine 4 histone 3
HSC: hepatic stellate cell
MFB: myofibroblasts
MTD: myofibroblast transdifferentiation
PPAR-γ: peroxisome proliferator-activated receptor gamma
RT-PCR: reverse-transcription polymerase chain reaction
αSMA: alpha smooth muscle actin
TGF-β1: transforming growth factor beta1
TIMP-1: tissue inhibitor of metalloproteinase-1
