# Supplementary material for: Histone Methyltransferase ASH1 Orchestrates Fibrogenic Gene Transcription During Myofibroblast Transdifferentiation
Source: Hepatology. 2012 Aug 28;56(3):1129–39. doi: 10.1002/hep.25754 (PMC3430805; doi:10.1002/hep.25754)
Supplement: Supplementary file 1 [file hep0056-1129-SD1.doc]

| **Gene** | **Alternative name** | **Forward and reverse primer pair sequences** | **Anneal temp** | **bp product** |
| --- | --- | --- | --- | --- |
| **SUV39H1** | KMT1A | ggcgccacctacctctttga | 54.6 | 124 |
|  |  | cgttgtacacctgcaggttg |  |  |
| **SUV39H2** | KMT1B | caaaaaggcacacagtattc | 48.4 | 119 |
|  |  | acctctccaacatattccat |  |  |
| **G9a** | KMT1C | gagccaccgagagagttc | 55.4 | 149 |
|  |  | ggtgtcagccccctcatc |  |  |
| **GLP** | KMT1D | acaaacagcgtggtcaag | 54.6 | 182 |
|  |  | tggaaacggtgagagatg |  |  |
| **SETDB1** | KMT1E | ctgggtatctctatggag | 49 | 195 |
|  |  | cttcaccaaagactcaca |  |  |
| **MLL1** | KMT2A | tctgtgttttcccctcta | 46.6 | 182 |
|  |  | ttcaggctatcttctttg |  |  |
| **MLL2** | KMT2B | aggagggcccagaactat | 53.4 | 177 |
|  |  | gcagtgttttcatggata |  |  |
| **MLL3** | KMT2C | cagccgcagacaaaagac | 49.7 | 177 |
|  |  | attgttctttgatttctg |  |  |
| **MLL5** | KMT2E | ccattgggggttgataca | 52.2 | 198 |
|  |  | aggaggacgagcaccata |  |  |
| **SET1a** | KMT2F | tctccagcagccgacaag | 53.2 | 143 |
|  |  | gaaggaggttgaagtggt |  |  |
| **NSD1** | KMT3B | tgccatcctcgagctgttcc | 53 | 158 |
|  |  | cccatcttatccttgctgct |  |  |
| **ASH1** | KMT2H | aatgatctttgctgagtgtt | 50.2 | 127 |
|  |  | tccccaacctttttcctcag |  |  |
| **SMYD2** | KMT3C | gccgtgagggagtttgagtc | 55.6 | 154 |
|  |  | agttcacctgggcaaagagc |  |  |
| **DOT1** | KMT4 | gtggcccagatgattgatga | 54.4 | 200 |
|  |  | ccataccatttcatccactt |  |  |
| **EZH2** | KMT6 | gttcgtgcccttgtgtgata | 53 | 145 |
|  |  | cactctcggacagccaggta |  |  |
| **SUV4-20H1** | KMT5B | aagggcagagtcgctatgtg | 55 | 178 |
|  |  | tagggttgttgtgagaaaaa |  |  |
| **SUV4-20H2** | KMT5C | gcgggaagaggatgaatacc | 55 | 166 |
|  |  | ttccatctgagggcacaaac |  |  |
| **LSD1** | KDM1 | tctcaggaagcagcgtgttt | 51.9 | 194 |
|  |  | ctatgaactcggtggacaag |  |  |
| **JMJD2A** | KDM4A | tcccaggaagtgctcaaagc | 54.2 | 205 |
|  |  | cgacgggtagcaaaattggt |  |  |
| **JMJD2B** | KDM4B | cgatggaaactgaaatgtgt | 54.7 | 177 |
|  |  | cttgaggcaggtgatggaca |  |  |
| **JMJD2C** | KDM4C | cagatggtcacagggcagtc | 52.4 | 227 |
|  |  | cattcatccacaccctcatc |  |  |
| **JMJD2D** | KDM4D | ccgatttatggtgctgacat | 50.6 | 152 |
|  |  | atgccaaagtacaagtaggg |  |  |
| **JARID1B** | KDM5B | caaaacgggaaaacgagaaa | 53.6 | 166 |
|  |  | tcattgccgctgccacataa |  |  |
| **JMJD3** | KDM6B | accaccacccaagaagagga | 57 | 176 |
|  |  | ccagtcccccgataacagta |  |  |
